# Adaptive Correlation Model for Visual Tracking Using Keypoints Matching and Deep Convolutional Feature

**DOI:** 10.3390/s18020653

**Published:** 2018-02-23

**Authors:** Yuankun Li, Tingfa Xu, Honggao Deng, Guokai Shi, Jie Guo

**Affiliations:** 1 School of Optics and Photonics, Image Engineering & Video Technology Lab, Beijing Institute of Technology, Beijing 100081, China; liyuankunbixian@gmail.com (Y.L.); dhg007@sina.com (H.D.); shi_guokai_123@126.com (G.S.); jieguo_2013@163.com (J.G.); 2 Key Laboratory of Photoelectronic Imaging Technology and System, Ministry of Education of China, Beijing 100081, China; 3 School of Information and Communication, Guangxi Key Laboratory of Wireless Wideband Communication and Signal Processing, Guilin University of Electronic Technology, Guilin 541004, China

**Keywords:** correlation filter-based visual tracking, deep convolutional neural network, deep convolutional feature, keypoints matching, adaptive model updating

## Abstract

Although correlation filter (CF)-based visual tracking algorithms have achieved appealing results, there are still some problems to be solved. When the target object goes through long-term occlusions or scale variation, the correlation model used in existing CF-based algorithms will inevitably learn some non-target information or partial-target information. In order to avoid model contamination and enhance the adaptability of model updating, we introduce the keypoints matching strategy and adjust the model learning rate dynamically according to the matching score. Moreover, the proposed approach extracts convolutional features from a deep convolutional neural network (DCNN) to accurately estimate the position and scale of the target. Experimental results demonstrate that the proposed tracker has achieved satisfactory performance in a wide range of challenging tracking scenarios.

## 1. Introduction

As one of the fundamental research topics in military, security, and human–computer interaction, visual tracking plays an important role in many applications. Given the initial motion state of the target object in the first frame, a visual tracking algorithm aims to estimate the motion state of the target object in each subsequent frame. Despite the massive work done in recent years, visual tracking is still challenging due to the appearance of variations caused by occlusion, target rotation, scale variation, and so on.

The correlation filter (CF) was originally designed to generate correlation peak output for an input signal. According to the convolution theorem, correlation operations can be significantly accelerated using fast Fourier transformation. In general, CF-based visual trackers use correlation filters to model the appearance of the target and update correlation filters at each frame using a fixed learning rate. However, due to the unreliable tracking caused by occlusion, background clutter, and other perturbations, correlation models may be contaminated during the updating process. In order to alleviate model contamination, dynamic adjustment of learning rate will be necessary and constructive.

To account for the target appearance changes over time, man kinds of feature descriptors have been used in visual tracking, such as Haar-like features [1], Color Names [2], FAST [3], and HOG [4]. Recently, features learned from deep convolutional neural networks (DCNNs) have been used in a variety of visual tasks. However, the outstanding performance of DCNNs relies heavily on training on large-scale datasets. Thus, the application of DCNNs in visual tracking is severely restricted by the very limited information in the first frame. Several tracking approaches [5,6] use pre-trained DCNNs as feature extractors. However, these approaches use only a few convolutional layers and cannot provide a comprehensive description of the target state.

Based on the discussion above, we propose a visual tracking framework that utilizes DCNN and CF synthetically. The main contributions of this paper are as follows:

1. We propose a novel model updating method. Firstly, we establish a keypoints library to restore the reliable historical data, and then we obtain the pixel-level correspondence between the current frame and the previous frame using dense matching. Finally, the similarity score is calculated by comparing matched pairs of keypoints and is used to adjust the learning rate in model updating.
2. We propose a method to fully exploit the hierarchical features generated of the DCNN, which can make full use of spatial detail information and semantic information.
3. Based on the observation of different layers’ output, we propose a scale estimation method using deep convolutional features.

The rest of the paper is organized as follows: In Section 2, we review research work related to ours. In Section 3, we present the proposed visual tracking framework in detail. Numerous experimental results and analysis are shown in Section 4. In Section 5, we reach the conclusions of our work.

## 2. Related Work

In this section, we list some works closely related to ours.

### 2.1. Trackers with Convolutional Neural Network

In recent years, convolutional neural networks (CNNs) have made significant progress on a wide range of computer vision issues, including visual tracking. Based on the combination of off-line pre-training and on-line fine-tuning, Wang et al. proposed the deep learning tracker (DLT) [7] and the structured output deep learning tracker (SO-DLT) [8] in the framework of particle filters. To avoid the issues caused by offline training, trackers in [9,10] incrementally learn target-specific CNNs without pre-training. The trackers mentioned above simply treat the CNN as a black-box classifier where only the outputs of the last layer are used to represent the target object. However, the goal of visual tracking is to estimate the target state precisely rather than to infer their semantic classes. Ma et al. [5] extract the hierarchical convolutional features (HCF) from three layers of CNN to learn multiple correlation filters for visual tracking. Danelljan et al. [11] proposed a tracker by learning continuous convolution operators (CCOT) to interpolate discrete features and train spatial continuous convolution filters, which enables efficient integration of multi-resolution deep feature maps. To alleviate the low computational efficiency caused by CNN operation, Danelljan et al. [12] designed an efficient convolution operators (ECO) for visual tracking using a factorized convolution operation. Although CCOT and ECO trackers use convolutional features for translation estimation, neither of them takes full advantage of the entire CNN and thus lack a complete description of the motion state of the target. Besides, compared with HCF tracker, CCOT and ECO trackers focus on the improvements to the CF model and ignore the problems during the model updating process.

### 2.2. Trackers with Correlation Filters

Since Bolme et al. [13] introduced correlation filters into visual tracking by minimizing the output sum of squared error (MOSSE), CF-based visual tracking algorithms have attracted considerable attention due to their high speed. Based on the raw pixel data, the MOSSE tracker performs high-speed CF training and tracking. The circulant structure and kernelized operator (CSK) [14] introduced in CF-based visual tracking algorithm significantly improves the capacity of the training set and thus improves the tracking accuracy. CSK was then extended to [2] and [4] by leveraging the HOG feature and the Color Names feature, respectively. Danelljan et al. [15] designed a scale estimation correlation filter to predict the spatial size of the target. In order to alleviate the boundary effect caused by circulant structure, Danelljan et al. [16] introduced spatial regularization in the cost function of correlation filters. By introducing mask matrix and sample cropping, Galoogahi et al. [16] alleviated the boundary effect in a different way. Based on the similarity between correlation and convolution operations, Valmadre et al. [17] construct a CNN where the correlation filter is part of the network and achieve end-to-end representation learning.

### 2.3. Trackers with Keypoints and Matching

Part-based visual tracking methods have exhibited outstanding performance against occlusion. While some trackers [18,19] choose rectangular parts as matching parts, the size and number of rectangular parts limit the speed of tracking. Instead, matching with feature point descriptors (such as SIFT [20] and BRISK [21]) is rather computationally convenient, which makes keypoints an ideal representation for modeling local part. In [22], Grabner et al. employed a boost classifier to obtain keypoints matching. Hare et al. [23] attached weights on different keypoints and update the weights in a unified framework. Tracker in [3] jointly uses optical flow tracking and keypoint matching to provide an estimate of both target position and target rotation.

## 3. Proposed Approach

**Figure 1:**
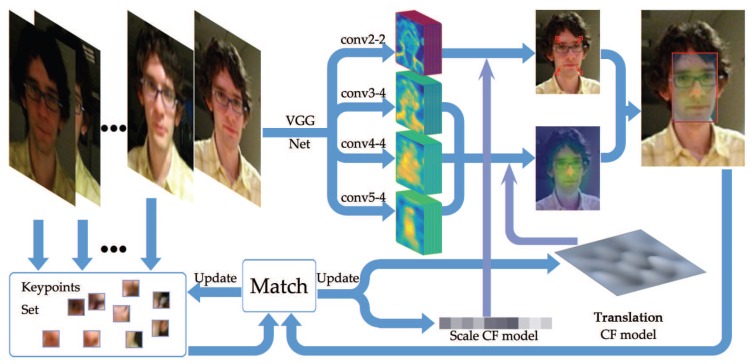
Flow chart of the proposed framework. CF: correlation filter.

Figure 1 shows an overall flow of the proposed ACMD (Adaptive Correlation model for visual tracking using keypoints Matching and Deep convolutional feature) tracker. An input image of the *t*-th frame is first pre-processed to fit the network input. Then, the conv2-2, conv3-4, conv4-4, and conv5-4 layers of VGG-19 [24] are used as feature extractors. These features are then convolved with two learned CF models to provide translation estimation and scale estimation. Final estimation of the *t*-th frame is achieved by the combination of two CF models’ output. A dense matching is then employed between current frame and previous frame and the matching score is used to update the keypoints set and discount the learning rate.

### 3.1. Deep Convolutional Features

**Figure 2:**
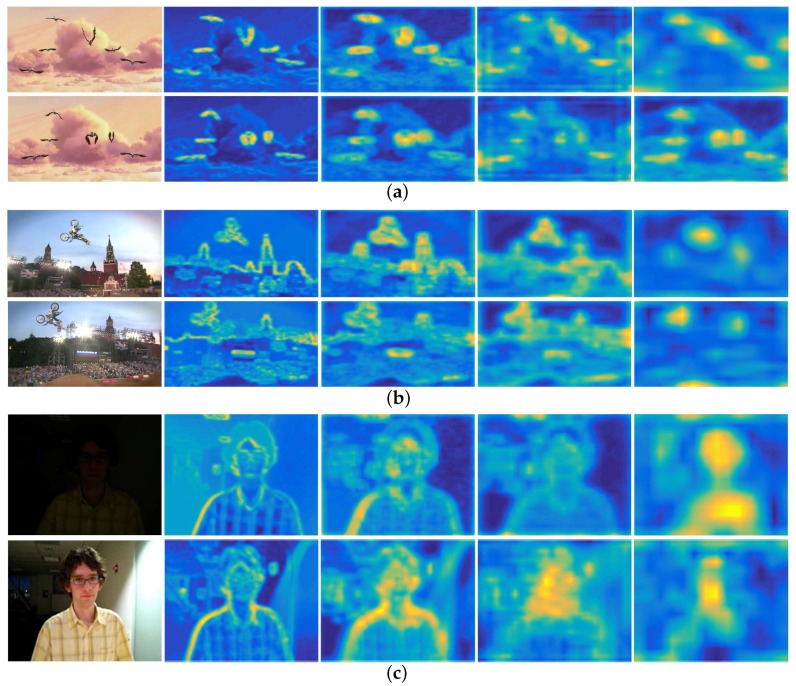
Visualization of input and outputs of different layers. From left to right are the input frame, feature maps of conv2-2, feature maps of conv3-4, feature maps of conv4-4, and feature maps of conv5-4. (**a**) Bird1; (**b**) MotorRolling; (**c**) David.

It should be noted that research [25] suggests that CNN’s improved performance is obtained using convolutional layers rather than fully-connected layers. Hence, we use convolutional layers to extract features. Recent studies [5,11,25] and our experimental results illustrate that:

1. CNN feature maps are high-dimensional features and contain information highly related to the target state.
2. Different layers of CNN encode different types of information. Feature maps of higher layers encode semantic information. As shown in Figure 2, although the appearance of the targets undergoes different variation (non-rigid deformation in Bird1, in-plane-rotation in MotorRolling, illumination variation in David), the region around the target is always bright yellow in feature maps of conv5-4 layer. This character is quite useful when the target undergoes severe appearance variation.
3. Feature maps of lower layers retain more spatial details of the target, such as borders, corners, and curves. Taking David as an example, it is obvious that the texture of the face such as edges and contours are well preserved, including the corner of the ear, the boundary of the face, etc., which could be used to determine the boundary of the target and thus to make scale estimation.

The net we employed is the VGG-19 [24] network, which was pre-trained offline using the ImageNet [26] dataset for classification tasks. It should be noted that the VGG-19 network takes 224 × 224 RGB images as input, so the input frame must be resized first. Additionally, on account of the pooling method, the spatial resolution decreases gradually as the net propagates forward, so an upsampling process is necessary after we extract raw convolutional features. We apply bilinear interpolation for both resizing and upsampling processes.

Let *x* denote the input RGB image patch and $f_{c}$ denote the feature map generated by the *c*-th convolutional layer. After the feature extraction procedure, $f_{c}$ shares the same spatial resolution with *x*, while the dimensionality of $f_{c}$ is determined by the value of *c*.

### 3.2. Correlation Filter

Traditionally, the goal of training is to find a correlation filter template *h* that minimizes the output of Equation (1) :

(1) $$\min_{h}{∥h^{T}f-g∥}^{2}+\lambda {∥h∥}^{2}.$$

Equation (1) is the form of the cost function of ridge regression, in which *f* is the training sample, *g* is the desired output, and λ≥0 is the regularization parameter. The superscript *T* denotes matrix transpose operation.

#### 3.2.1. Correlation Filters for Translation Estimation

It should be noted that only one training sample in one dimensionality is taken into consideration in Equation (1). A circulant structure is used to generate a set of training samples $\left\{f_{m,n}|\left(m,n\right)\in \left\{0,1\ldots ,M\right\}\times \left\{0,1\ldots ,N\right\}\right\}$. Equation (1) can be transformed into the following form:

(2) $$\min_{h}\sum_{m,n}{\left|\left|\sum_{l=1}^{L}{\left(h_{m,n}^{l}\right)}^{T}f_{m,n}^{l}-g\left(m,n\right)\right|\right|}^{2}+\lambda \sum_{l=1}^{L}{∥h^{l}∥}^{2},$$

where superscript *l* denotes the *l*-th dimensionality of a matrix and *g*(*m*,*n*) is the GAUSSIAN shaped label:

(3) $$g\left(m,n\right)=exp\left[-\frac{{\left(m-M/2\right)}^{2}+{\left(n-N/2\right)}^{2}}{2\sigma^{2}}\right].$$

According to [4], the solution to Equation (2) is:

(4) $$H^{l}=\frac{G★\bar{F^{l}}}{\sum_{i=1}^{L}F^{i}★\bar{F^{i}}+\lambda },$$

where capital letters denote the Fourier transformation form, the overbar notation denotes complex conjugation form, and the ★ operator performs an element-wise multiplication of the two matrices.

Given a sample patch $f_{c}$, the correlation response output $y_{c}$ is calculated by Equation (5):

(5) $$y_{c}=F^{-1}\left(\sum_{l=1}^{L}H^{l}★\bar{{f_{c}}^{l}}\right),$$

where $F^{-1}$ denotes the inverse Fourier transform operator. The final response output for translation estimation is obtained by a weighted average of all $y_{c}$:

(6) $$y_{trans}=\frac{\sum_{c}\left(\mu_{c}y_{c}\right)}{\sum_{c}\mu_{c}}.$$

The new target center is estimated to be at the position of maximum value of $y_{trans}$.

#### 3.2.2. Correlation Filters for Scale Estimation

The correlation filter for scale estimation can also be obtained by the minimization Equation (1). Note that in scale space, samples expand in one dimension, so the set of training samples is generated like $\left\{f_{s}|\left(s\right)\in \left\{0,1\ldots ,S\right\}\right\}$, and label in GAUSSIAN shaped $g\left(s\right)$ is as follows:

(7) $$g\left(s\right)=exp\left[-\frac{{\left(s-S/2\right)}^{2}}{2\sigma^{2}}\right].$$

Equation (1) can be can be rewritten as follows:

(8) $$\min_{h}\sum_{s}{\left|\left|\sum_{l=1}^{L}{\left(h_{s}^{l}\right)}^{T}f_{s}^{l}-g\left(s\right)\right|\right|}^{2}+\lambda \sum_{l=1}^{L}{∥h_{s}^{l}∥}^{2}.$$

The solution to Equation (8) is same as Equation (4), and the correlation response output can be calculated by:

(9) $$y_{scale}=F^{-1}\left(\sum_{l=1}^{L}H^{l}★\bar{{f_{s}}^{l}}\right).$$

The current scale shares the same coordinate index with the maximum value of $y_{scale}$.

### 3.3. Adaptive Model Updating

The correlation model learned at the *t*-th frame $H_{t}^{l}$ can be expressed in fractional form. Conventionally, the numerator $A_{t}^{l}$ and denominator $B_{t}$ of the correlation filter are updated using fixed learning rate η:

(10) $$A_{t}^{l}=\left(1-\eta_{t}\right)A_{t-1}^{l}+\eta_{t}\left({\bar{G}}_{t}★F_{t}^{l}\right),$$

(11) $$B_{t}^{l}=\left(1-\eta_{t}\right)B_{t-1}^{l}+\eta_{t}\left(\sum_{i=1}^{L}F^{i}★\bar{F^{i}}\right).$$

It is not difficult to conclude that when the *T*-th frame arrives, the contribution rate of the *t*-th frame (1<t<T) to the model is: ${\left(1-\eta \right)}^{T-t}\eta$. This can lead to two problems. First, the unreliable tracking data of the *t*-th frame will affect the tracking results of all subsequent frames. Second, when long-term occlusion occurs, continuous learning of corrupt data will force the correlation model to fit the occlusion information and thus reduce the discrimination of the model. To alleviate these two problems, we propose an adaptive model updating method.

Let $K_{t-1}$ denote the keypoints set established in the 1st ~$\left(t-1\right)$-th frames and $D_{t-1}$ denote the corresponding set of descriptors of $K_{t-1}$. We initialize $K_{1}$ and $D_{1}$ at the first frame using the FAST [27] detector and the BRISK [21] descriptor. Firstly, the pixel-level correspondence between current *t*-th frames and the t−1-th frame is obtained using dense matching; that is, finding the matched point $k_{curr}$ in the current frame for $k_{t-1}\in K_{t-1}$.

(12) $$k_{curr}=M\left(k_{t-1}\right),$$

(13) $$d_{curr}=D\left(k_{curr}\right).$$

where M is the dense matching process and D is the descriptor calculation process. We use the Hamming distance to define the similarity between $k_{curr}$ and $k_{t-1}$:

(14) $$V\left(k_{t-1},k_{curr}\right)=1-\frac{\sum_{i}k_{t-1}\left[i\right]⊕d_{curr}\left[i\right]}{v_{max}}.$$

where *i* is the index of sub-element in the descriptor and $v_{max}$ is the maximum Hamming distance. ⊕ denotes the exclusive-OR operation. In our case, $v_{max}$ is equal to 512. Let $K_{t-1}^{sub}$ denote the set of points in the $K_{t-1}$ that participate in the dense matching process, $K_{t-1}^{sub}\subset K_{t-1}$. Additionally, let $K_{curr}$ denote the set of points matched in the *t*-th frame. Then, the matching similarity score is calculated as follows:

(15) $$\rho_{t}=\frac{1}{N}\sum V\left(K_{t-1},K_{curr}\right).$$

where $V\left(K_{t-1},K_{curr}\right)$ denotes the similarities of each element in set $K_{t-1}$ and the corresponding element in set $K_{curr}$.

The learning rate $\eta_{t}$ consists of two parts: the basic learning rate $\eta_{0}$ and the discounting factor $\rho_{t}$ :

(16) $$\eta_{t}=\eta_{0}\rho_{t}.$$

Since both Equation (14) and Equation (15) are normalized, the matching similarity score in Equation (15) can be used as the discount factor directly.

The the feature point library can be updated as follows: for $k_{curr}$, if the similarity $V\left(k_{t-1},k_{curr}\right)$ between $k_{curr}$ and $k_{t-1}$ is larger than a threshold, use $u_{curr}$ as $u_{t}$; otherwise, use $u_{t-1}$ as $u_{t}$.

(17) $$\left\{k_{t},d_{t}\right\}=\left\{\begin{array}{c} \left\{k_{curr},D\left(k_{curr}\right)\right\},V\left(k_{t-1},k_{curr}\right)\geq v_{thresh}\\ \left\{k_{t-1},d_{t-1}\right\},V\left(k_{t-1},k_{curr}\right)<v_{thresh} \end{array}\right.$$

The overall tracking algorithm is described in Algorithm 1.

**Algorithm 1:** Proposed tracking algorithm.**Input :** Image *I*; initial target position $p_{0}$ and scale $s_{0}$; previous target position $p_{t-1}$ and scale $s_{t-1}$.**Output :** Estimated object position $p_{t}$ and scale $s_{t}$.
Initialize correlation filters $H_{1}^{trans}$, $H_{1}^{scale}$ and set $K_{1}$, $D_{1}$**Foreach**
$I_{t}$ Extract multiple $f_{c}$ generated by VGG-Net; Compute the translation correlation $y_{trans}$ using Equation (5) and Equation (6) Set $p_{t}$ to at the maximum of $y_{trans}$ Compute the translation correlation $y_{trans}$ using Equation (9) Set $s_{t}$ to at the maximum of $y_{scale}$ Compute discounting factor $\rho_{t}$ using Equation (12) to Equation (15) Update $A_{trans}$, $B_{trans}$, $A_{scale}$, $B_{scale}$, *K*, *D***End**

## 4. Experiments

We evaluated the performance of our approach on the OTB-2013 [28] dataset and the OTB-2015 [29] dataset and compared our approach with 10 state-of-the-art trackers, including HCF [5], SRDCFad [30], SCT [31], MEEM [32], SAMF [33], DSST [15], KCF [4], STRUCT [34], TLD [35], SCM [36], and DLT [7]. Among those trackers, HCF and DLT use DCNN; HCF, SRDCFad, SCT, SAMF, DSST, and KCF are CF-based trackers and SRDCFad proposes an adaptive model updating method.

We implemented the proposed tracker in MATLAB 2015b. All of the experiments were performed on a PC with an Intel i7-4790 CPU. The speed of all trackers is shown in Table 1.

The parameters, which are fixed for each sequence, are summarized as follows. The net employed for feature extraction was a pretrained version of VGG-19 [24]. We extracted the feature maps from the conv2-2, conv3-4, conv4-4, and conv5-4 layers. The basic learning rate $\eta_{0}$ in Equation (16) was set to 0.025. To make scale estimation, we set S=15 and set the scale stride to 1.04. The weight in Equation (6) was set to $\mu_{5\_4}=0.44,\mu_{4\_4}=0.33,\mu_{3\_4}=0.23$. The dimensionality of keypoints descriptor was set to 512, and the threshold in Equation (17) was set to 300.

### 4.1. Quantitative Evaluation

We used the precision and success rate as the evaluation criteria of quantitative analysis. The precision criteria measure Euclidean distance between the center of tracker’s output and the ground truth. Precision plots show the percentage of frames whose precision is greater than a threshold. According to Reference [28], we used a threshold of 20 pixels. Another criteria—success rate—measures the coverage between tracker’s output and the ground truth. Assuming that the region of the tracker’s output is $\gamma_{t}$ and the region of ground truth is $\gamma_{a}$, the success rate is defined as:

(18) $$SuccessRate=\frac{\left|\gamma_{t}\cap \gamma_{a}\right|}{\left|\gamma_{t}\cup \gamma_{a}\right|},$$

where ∩ and ∪ denote the intersection and union of two regions, respectively, and $\left|\cdot \right|$ denotes the number of pixels in the region. The success plot illustrates the percentage of frames whose success rates are greater than a certain value. According to [28], we ranked all trackers using area under the curve (AUC) for the success rate.

**Figure 3:**
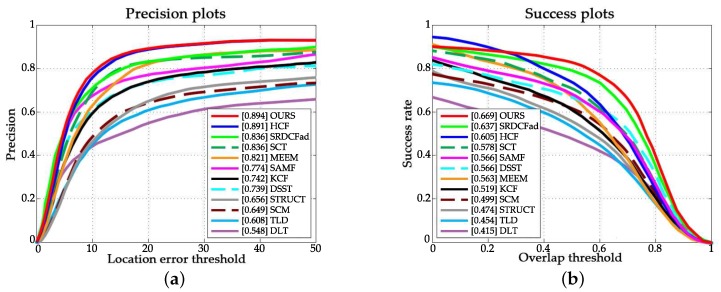
Precision and success plots over the OTB-2013 dataset. (**a**) Precision plot; (**b**) Success plots.

**Figure 4:**
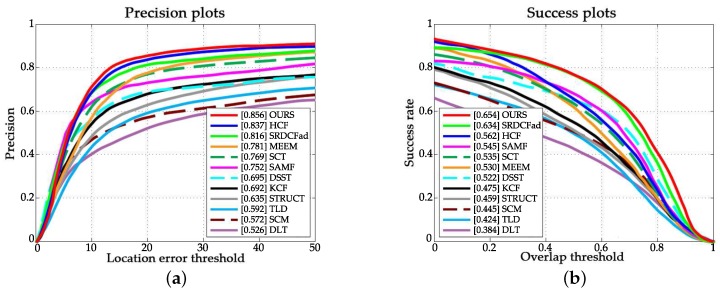
Precision and success plots over the OTB-2015 dataset. (**a**) Precision plot; (**b**) Success plots.

Figure 3 and Figure 4 illustrate the overall performance of all trackers in terms of the mentioned criteria. The proposed approach ranks first over both OTB-2013 and OTB-2015.

**Figure 5:**
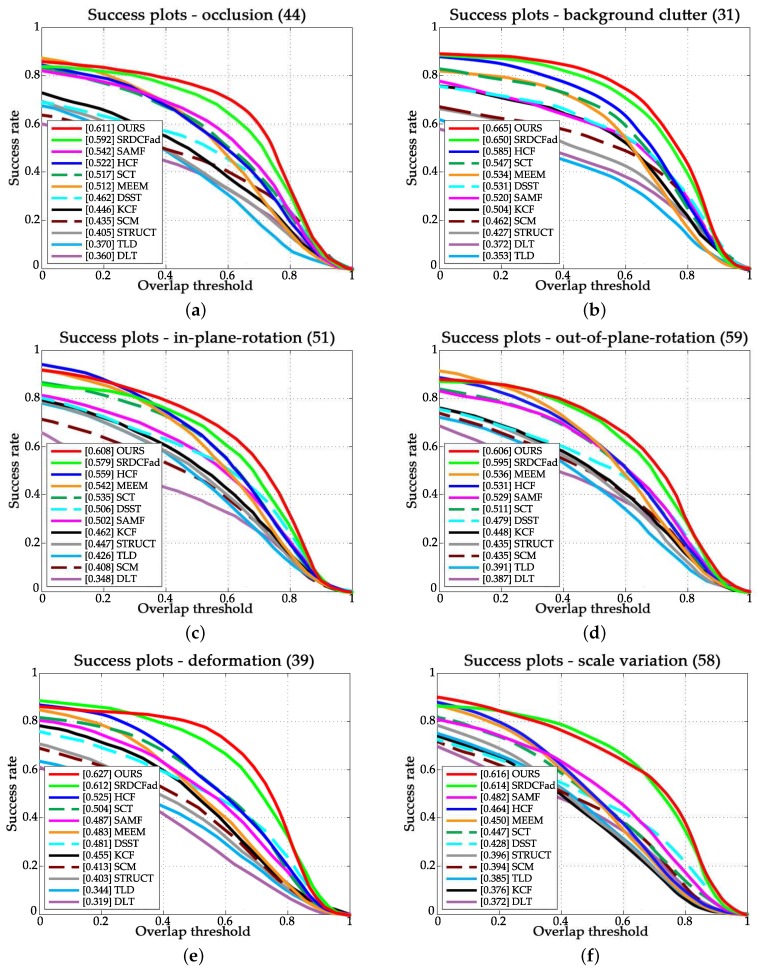
Success plots over six tracking challenges of (**a**) occlusion; (**b**) background clutter; (**c**) in-plane rotation; (**d**) out-of-plane; (**e**) deformation; and (**f**) motion blur.

Attribute-based experimental results are shown in Figure 5. From Figure 5, we have the following observations. Firstly, our approach handled occlusion efficiently, which can be explained by the proposed adaptive model updating method. This method also helped to improve the performance in sequences with attribute of background cluster. Secondly, our approach performed well in the sequences with attributes of rotation and deformation, as the higher layers of CNN retain rich semantic information of the target object. Thirdly, our approach performs favorably against other approaches in sequences with the attribute of scale variation due to the rich texture information encoded in lower layers.

### 4.2. Qualitative Evaluation

To better analyze the effectiveness and robustness of the proposed tracker, this section is divided into two subsections to conduct a qualitative analysis.

#### 4.2.1. Performance against Background Information Variation

**Figure 6:**
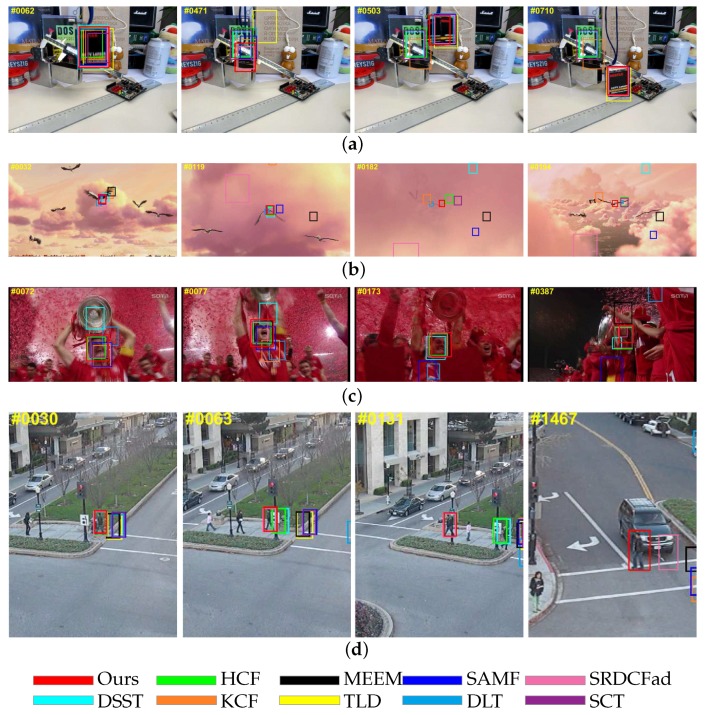
Tracking results on sequences with attributes of occlusion and background cluster. From top to bottom, the name of the video is (**a**) Box; (**b**) Bird1; (**c**) Soccer; (**d**) Human3.

In this section, we focus on the trackers’ performance against background information variation, including occlusion and background cluster. Figure 6 shows the situation in which the target undergoes severe occlusion and background cluster. In the sequence box and Bird1, the occlusion takes up to 35 frames and 50 frames, respectively. In the sequence Human3, the target is occluded by two different objects. In the sequence Soccer, spatial context information of the target changes dramatically and the target is nearly fully occluded. Due to the proposed updating method, the discounting factor decreases to near zero when the target undergoes occlusion, which prevents the correlation filter from learning occlusion information and losing the ability to discriminate the target. It should be noted that in the sequence Human3, only our tracker and SRDCFad succeeded in tracking the target at the first 1400 frames, which means that an adaptive model updating method is significant when the occlusion situation is complex. Besides, semantic information encoded in higher layers ensures that the tracker is not sensitive to background cluster.

#### 4.2.2. Performance against Target Appearance Variation

**Figure 7:**
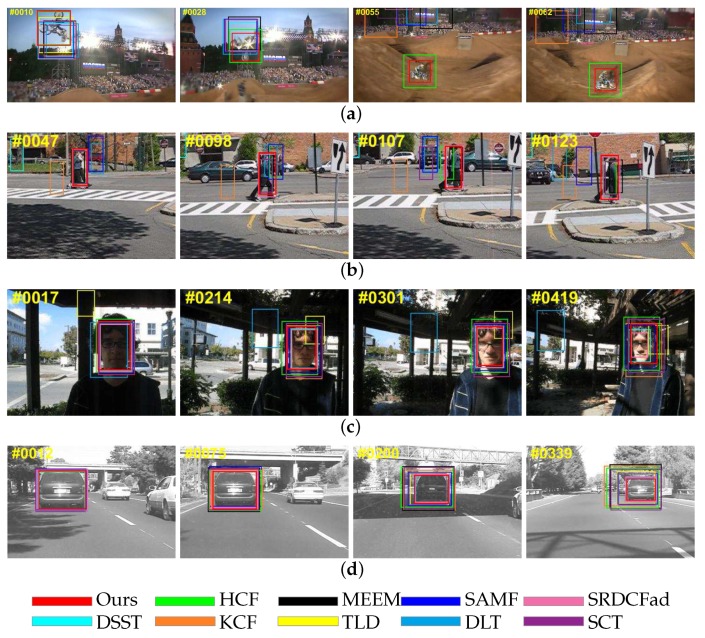
Tracking results on sequences with attributes of occlusion and background cluster. From top to bottom, the name of the video is (**a**) MotorRolling; (**b**) Couple; (**c**) Trellis; (**d**) Car4.

In this section, we will discuss the trackers’ performance against the variation of target appearance. In Figure 7a, the near-270-degree in-plane rotation of motorcyclists in the MotorRolling sequence is a big challenge for visual tracking algorithms. Since the high layers of CNN retain rich semantics information, our tracker performed well in this sequence. Although HCF and DLT also use convolutional features, these trackers cannot fully exploit fine-grained information, so HCF failed in scale estimation and DLT lost target totally. Similar results also appeared in the sequence Couple with the attribute of out-of-plane rotation (Figure 7b). In the sequences Trellis (Figure 7c) and Car4 (Figure 7d), the targets undergo scale variation and illumination variation at the same time, making it difficult to determine the precise boundary of the target. Since the fine-grained information in the lower layers is used properly, our tracker provided accurate scale estimation in these two sequences.

### 4.3. Demonstrations

To evaluate the effect of updating method and scale estimation, we conducted additional comparison experiments on the OTB-2015 dataset.

#### 4.3.1. Evaluation of the Updating Method

We compared our method with the updating method using peak to sidelobe ratio (PSR) and updating method using fixed learning rate. The calculation of PSR is described in [13]. Let $PSR_{mean}$ denote the average value of the historical data of the PSR. The application of PSR can be can be expressed by Equation (19):

(19) $$\eta =\left\{\begin{array}{c} 0,PSR<PSR_{mean}-2\\ \eta ,PSR\geq PSR_{mean}-2 \end{array}\right..$$

**Figure 8:**
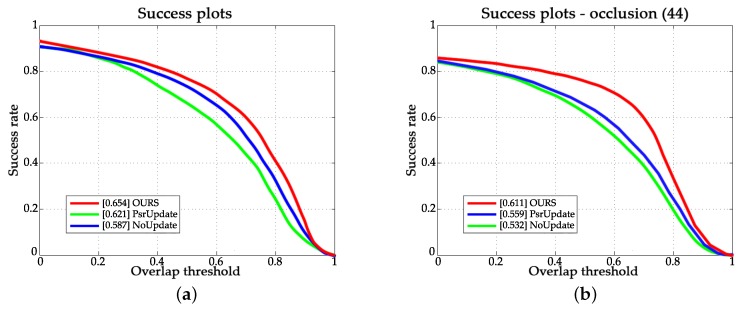
Tracking performance of different updating methods. (**a**) Success plots on OTB-2015; (**b**) Success plots over sequences with occlusion attribute.

Experimental results are shown in Figure 8. ACMD is our proposed method. PsrUpdate is the same as ACMD, except it uses PSR as an update criterion. No update indicates that it uses a fixed learning rate update method. As shown in Figure 8a, ACMD led to 6.7% performance improvements in terms of success rate on OTB-2015 dataset. Besides, from Figure 8 we can find that the improvement of using PSR is limited. Moreover, as we can easily see in Figure 8b, the ACMD had a greater advantage over sequences with occlusion attribute, which illustrates the effectiveness of the proposed updating method in handling occlusion issues.

#### 4.3.2. Evaluation of Scale Estimation

**Figure 9:**
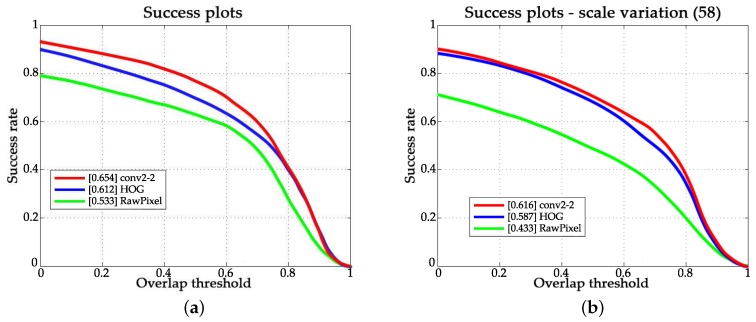
Scale estimation performance using different features. (**a**) Success plots on OTB-2015; (**b**) Success plots over sequences with the attribute of scale variation.

In order to evaluate the performance of convolutional features for scale estimation, we used the HOG feature and the raw pixel feature as comparisons. The results are shown in Figure 9. The legend in Figure 9 annotates the feature used in the corresponding curve. From Figure 9, we can see that scale estimation using convolution feature works best in scale estimation. In the success rate evaluation over all 100 sequences and 58 sequences with attribute of scale variation, convolutional feature-based scale estimation led to 23.9% and 19.7% performance improvement, respectively.

### 4.4. Failure Cases

**Figure 10:**
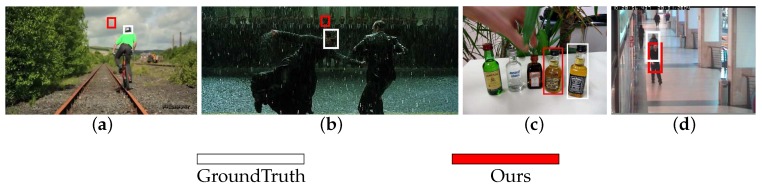
Failure cases on the following sequences: (**a**) Biker; (**b**) Matrix; (**c**) Liquor; (**d**) Walking2.

We show a few failure cases in Figure 10. For the Biker and Matrix sequences, when the target object undergoes fast motion, the proposed tracker fails to follow targets due to the boundary effect introduced in the correlation model. For the Liquor and Walking2 sequences, the proposed method fails to track the target as the target is occluded by a similar object and the the discounting factor cannot decrease when occlusion occurs. Correlation filters with less boundary effect and strategies for handling similar object interference will be considered in our future work.

## 5. Conclusions

In this paper, we propose a visual tracking framework which synthesizes features from multiple layers in a CNN and makes full use of the VGG network. The proposed tracker can make precise position estimation in many challenging videos. The novel model updating method of the tracker improves the tracking performance in occlusion scenarios. Moreover, the use of convolutional features ensures the accuracy of scale estimation. Numerous experimental results demonstrate that the proposed tracker outperforms the state-of-the-art trackers in both precision and success rate.

## Figures and Tables

**Table 1:** Speed of the trackers.

	Ours	SRDCFad	HCF	SCT	MEEM	SAMF	DSST	KCF	STRUCT	TLD	SCM	DLT
Average FPS	3	3	6	44	11	12	56	192	10	22	0.4	0.6
